# Singaporean Mothers’ Perception of Their Three-year-old Child’s Weight Status: A Cross-Sectional Study

**DOI:** 10.1371/journal.pone.0147563

**Published:** 2016-01-28

**Authors:** Tuck Seng Cheng, See Ling Loy, Yin Bun Cheung, Jerry Kok Yen Chan, Mya Thway Tint, Keith M. Godfrey, Peter D. Gluckman, Kenneth Kwek, Seang Mei Saw, Yap-Seng Chong, Yung Seng Lee, Fabian Yap, Ngee Lek, Growing Up in Singapore Towards Healthy Outcomes (GUSTO) study group

**Affiliations:** 1 Department of Paediatrics, KK Women’s and Children’s Hospital, Singapore, Singapore; 2 KK Research Centre, KK Women’s and Children’s Hospital, Singapore, Singapore; 3 Center for Quantitative Medicine, Duke-NUS Medical School, Singapore, Singapore; 4 Department of International Health, University of Tampere, Tampere, Finland; 5 Duke-NUS Medical School, Singapore, Singapore; 6 Department of Reproductive Medicine, KK Women’s and Children’s Hospital, Singapore, Singapore; 7 Department of Obstetrics and Gynaecology, Yong Loo Lin School of Medicine, National University of Singapore, National University Health Systems, Singapore, Singapore; 8 MRC Life-course Epidemiology Unit, University of Southampton, Southampton, United Kingdom; 9 NIHR Southampton Biomedical Research Centre, University of Southampton and University Hospital Southampton NHS Foundation Trust, Southampton, United Kingdom; 10 Liggins Institute, University of Auckland, Auckland, New Zealand; 11 Singapore Institute for Clinical Sciences, Agency for Science, Technology and Research, (A*STAR), Singapore, Singapore; 12 Department of Maternal Fetal Medicine, KK Women’s and Children’s Hospital, Singapore, Singapore; 13 Saw Swee Hock School of Public Health, National University of Singapore, Singapore, Singapore; 14 Department of Paediatrics, Yong Loo Lin School of Medicine, National University of Singapore, National University Health Systems, Singapore, Singapore; Institute of Preventive Medicine, DENMARK

## Abstract

**Objective:**

Inaccurate parental perception of their child’s weight status is commonly reported in Western countries. It is unclear whether similar misperception exists in Asian populations. This study aimed to evaluate the ability of Singaporean mothers to accurately describe their three-year-old child’s weight status verbally and visually.

**Methods:**

At three years post-delivery, weight and height of the children were measured. Body mass index (BMI) was calculated and converted into actual weight status using International Obesity Task Force criteria. The mothers were blinded to their child’s measurements and asked to verbally and visually describe what they perceived was their child’s actual weight status. Agreement between actual and described weight status was assessed using Cohen’s Kappa statistic (κ).

**Results:**

Of 1237 recruited participants, 66.4% (n = 821) with complete data on mothers’ verbal and visual perceptions and children’s anthropometric measurements were analysed. Nearly thirty percent of the mothers were unable to describe their child’s weight status accurately. In verbal description, 17.9% under-estimated and 11.8% over-estimated their child’s weight status. In visual description, 10.4% under-estimated and 19.6% over-estimated their child’s weight status. Many mothers of underweight children over-estimated (verbal 51.6%; visual 88.8%), and many mothers of overweight and obese children under-estimated (verbal 82.6%; visual 73.9%), their child’s weight status. In contrast, significantly fewer mothers of normal-weight children were inaccurate (verbal 16.8%; visual 8.8%). Birth order (p<0.001), maternal (p = 0.004) and child’s weight status (p<0.001) were associated with consistently inaccurate verbal and visual descriptions.

**Conclusions:**

Singaporean mothers, especially those of underweight and overweight children, may not be able to perceive their young child’s weight status accurately. To facilitate prevention of childhood obesity, educating parents and caregivers about their child’s weight status is needed.

## Introduction

The worldwide prevalence of overweight and obesity among preschool children has increased from 4.2% in 1990 to 6.7% in 2010 [1]. A further increase to 9.1% by 2020 has been projected unless effective interventions in early infancy are undertaken [1]. A recent study conducted among Singaporean Chinese preschoolers found a similarly high prevalence of overweight and obesity ranging from 7.0% to 8.1% [2]. These trends are noteworthy because pediatric obesity is associated with psychological morbidities and physical health issues including glucose intolerance, dyslipidaemia, hypertension, and cardiovascular risk [3–5]. Overweight children also tend to become overweight adults [6], which subsequently increase the risk of developing metabolic diseases [7, 8]

It has been proposed that the long term impact of childhood obesity on metabolic diseases could be avoided if early intervention to reduce body mass index (BMI) is undertaken [9]. Since parents play a key role in providing a young child’s contextual environment, active engagement of the parents constitutes an essential strategy in the prevention of childhood overweight and obesity [10]. Mothers are typically the primary caregivers who are able to influence the amount and type of food consumed by their child through encouragement or restriction, especially at younger age. They also play an important role in seeking medical assistance and advice if their child deviates from a healthy weight. Importantly, parents have to accurately recognize their child’s weight status in order for them to make appropriate lifestyle choices and adopt the correct health-seeking behaviours. Conversely, parental misperception can distort parental feeding practice and delay intervention, leading to an unhealthy body weight in the child [11].

A systematic review reported that parents demonstrated poor ability in accurately recognizing their child’s actual weight status [12]. Most of the studies, however, were conducted in western countries. As Asian economies develop, the prevalence of obesity and its associated complications such as diabetes and cardiovascular diseases is also rapidly increasing. Parental perceptions of child’s weight status in the Asian context may differ from those found in the West because of different socio-cultural backgrounds. A recent study did conclude that misperceptions of their 10- to 15-year old adolescents’ weights were prevalent among Chinese parents in Southern China [13]. However, there is no Asian report to-date on parental perceptions of toddlers and young children’s weight status. This study aimed (a) to evaluate the ability of Singaporean mothers in recognizing their child’s weight status at three years old using verbal and visual descriptions, (b) to assess the differences between verbal and visual descriptions, and (c) to identify factors associated with inaccurate weight perceptions by the mothers in their verbal and visual descriptions.

## Methods

### Study participants

Participants were drawn from an ongoing prospective mother-offspring cohort study, the Growing Up in Singapore Towards healthy Outcomes (GUSTO) [14]. A total of 1237 pregnant women who were aged 18 years and above and conceived naturally (n = 1152) or through *in vitro* fertilization (IVF) (n = 85) were recruited during the first trimester of pregnancy (<14 weeks of gestational age based on a dating ultrasound scan) at two major hospitals in Singapore (KK Women’s and Children’s Hospital and National University Hospital) in 2009 and 2010. The participants are Singapore citizens or permanent residents and have homogeneous parental ethnic groups (Chinese, Malays or Indians). Interviewer-administered questionnaires were completed at the recruitment visit and at 26–28 weeks of gestation to capture maternal socio-demographic characteristics. This study was approved by Domain Specific Review Board of the Singapore National Healthcare Group and the Centralised Institutional Review Board of SingHealth, and is conducted in accordance to Good Clinical Practice. All parents provided written informed consent for themselves and their child to participate in the cohort study. For the present analysis, we included all singleton children with complete actual weight status and whose mothers’ undertook the verbal and visual weight description tasks at 36 months post-delivery.

### Anthropometric measurements

All anthropometric measurements were recorded by trained clinical staffs using standardized techniques [15]. Mothers’ weight and height were measured at 18 months postpartum, and children’s weight and height were measured at 36 months of age. Height was measured twice to the nearest 0.1 centimetre (cm) using a portable stadiometer (SECA 213, Hamburg, Germany). Weight was measured twice to the nearest 0.1 kilogram (kg) using an electronic flat scale (SECA 803, Hamburg, Germany). The measurements were repeated when there was a difference in between the two readings of more than 1 cm in height and 0.2 kg in weight, respectively. The average of two nearest readings was used.

Body Mass Index (BMI) was calculated as weight (kg) divided by height squared (m^2^). The children’s actual weight status was defined according to the age- and gender-specific International Obesity Task Force (IOTF) BMI criteria, as: i) very underweight, ii) underweight, iii) normal, iv) overweight and v) obesity [16, 17]. For comparison with visual description, the children’s actual weight status were further re-classified into three categories: i) underweight (comprising very underweight and underweight), ii) normal (comprising normal) and iii) overweight (comprising overweight and obesity). For the main analyses of this study, the IOTF criteria were used to classify the children’s actual weight status because data from Singapore were included in the derivation of the IOTF references [16, 17]. Nevertheless, supplementary analyses were performed using the World Health Organization (WHO) standards [15], in which the children’s actual weight status were re-classified based on BMI-for-age Z-scores (BAZ) computed using WHO Anthro software (Version 3.2.2).

### Maternal perceptions of child’s weight status

Maternal perceptions of child’s weight status were assessed using a verbal description task and a visual description task, both administered at 36 months post-delivery.

#### 1) Verbal description

Mothers were asked to select the answer to the question “What do you think of your child’s weight?” from five choices (very underweight, underweight, normal, overweight or very overweight) which best described their child’s actual weight status. The selected answer was taken as the mothers’ verbal description of their child’s weight.

#### 2) Visual description

Mothers were shown the seven sketches of the previously validated Toddler Silhouette Scale (TSS) [18], randomly and one at a time, and asked to select the one which they thought most closely resembled their child’s prevailing weight status (written permission was obtained from the authors for use of TSS) [18]. The selected sketch was taken as the mothers’ visual description of their child’s weight, as follows: silhouette 1 depicts an underweight child, silhouettes 2, 3, 4 and 5 depict a normal-weight child, and silhouette 6 and 7 depict an overweight child, respectively (Fig 1 of Hager et al. [18]).

### Statistical analyses

Children’s actual weight status was cross-tabulated with mothers’ verbal or visual description. The degree of accuracy of mothers in perceiving their child’s weight status was stratified into: a) accurate perception, defined as mother’s description matching with their child’s actual weight status; b) underestimation, defined as mother’s description of child’s weight as lower than the child’s actual weight status; and c) overestimation, defined as mother’s description of child’s weight as higher than their child’s actual weight status. The agreements between perceived and actual weight status in the children based on the IOTF references were tested using Cohen’s Kappa statistic (κ). As supplementary analysis, the same test was repeated using actual weight status that was re-classified based on the WHO standards. Fisher’s exact tests for categorical variables and One-way Analysis of Variance (ANOVA) with post-hoc tests for continuous variables were performed to examine the factors that were associated with consistent and inconsistent misperceptions in the two tasks. Statistical significance was set at *p*<0.05. All statistical analyses were performed using Statistical Package for the Social Sciences, Version 19.0 (SPSS Inc. Chicago, Illinois, US).

## Results

A total of 821 (66.4%) mother-child pairs were included in this analysis. No significant differences were found between the excluded and included GUSTO participants in the type of conception, ethnicity, mother’s education level and BMI at 18 months post-delivery as well as the child’s gender and weight status and BMI at three years old. However, the mothers who were excluded tended to be single (5.2% vs 2.8%, p = 0.05), younger [mean 29.67 (SD 5.25) vs 31.04 (5.07) years old, p<0.001], did not attain tertiary education (36.5% vs 28.6%, p = 0.002) and had monthly household income of <SGD 2000 (17.7% vs 14.7%, p = 0.006), when compared to the mothers who were included (S1 Table).

At three years of age, majority of the children were normal weight (n = 591, 72.0%), followed by underweight (n = 150, 18.3%), overweight (n = 45, 5.5%), obese (n = 24, 2.9%) and very underweight (n = 11, 1.3%) children.

### Verbal and visual description of child’s weight status

Table 1 presents the agreement between mothers’ verbal description of their child’s weight status and the children’s actual weight status at 3 years old. The agreement between perceived and actual child’s weight status was fair (κ = 0.308). None of the 11 mothers whose child was very underweight could accurately describe their child’s weight status. Eight (72.7%) of the mothers of very underweight children described their child as underweight, whereas the other three mothers (27.3%) erroneously described their child as having normal weight. Among underweight children, more than half were inaccurately described as having normal weight (n = 72, 48.0%) and very underweight (n = 5, 3.3%). Majority of the normal weight children (n = 492, 83.2%) were accurately described while the remaining were either underestimated (n = 85, 14.4%) or overestimated (n = 14, 2.4%) by their mothers. High prevalence of weight underestimation was observed among overweight and obese children. About three quarters of mothers (n = 34, 75.6%) inaccurately described their overweight child as having normal weight. Only one of 24 mothers (4.2%) of obese children described her child accurately as very overweight; the other 23 mothers underestimated their obese child as overweight (n = 19, 79.2%) or normal weight (n = 4, 16.7%).

**Table 1:** The agreement between mother’s verbal description of the child’s perceived weight status and the child’s actual weight status based on the IOTF criteria [16, 17] at age 3 years.

Verbal description	Child’s actual weight status, n (%)					Kappa, κ
	Very underweight	Underweight	Normal	Overweight	Obesity	
	(n = 11)	(n = 150)	(n = 591)	(n = 45)	(n = 24)	
Very underweight	0 (0.0)	5 (3.3)	1 (0.2)	0 (0.0)	0 (0.0)	
Underweight	8 (72.7)	73 (48.7)	84 (14.2)	0 (0.0)	0 (0.0)	
Normal	3 (27.3)	72 (48.0)	492 (83.2)	34 (75.6)	4 (16.7)	0.308
Overweight	0 (0.0)	0 (0.0)	14 (2.4)	11 (24.4)	19 (79.2)	
Very overweight	0 (0.0)	0 (0.0)	0 (0.0)	0 (0.0)	1 (4.2)	

n = number; % = percentage

Table 2 presents the agreement between mothers’ visual description of their child’s weight status and the children’s actual weight status at 3 years old. Cohen’s Kappa test showed poor agreement between the perceived and actual child’s weight status (κ = 0.134). A substantial proportion of the mothers (n = 539, 91.2%) whose child’s actual weight status was normal accurately described their child’s weight status visually. However, 88.8% (n = 143) of the mothers of very underweight/underweight children overestimated their child’s weight status as normal or overweight, and 73.9% (n = 51) of the mothers of overweight/obese children underestimated their child’s weight as normal.

**Table 2:** The agreement between mother’s visual description of the child’s perceived weight status and the child’s actual weight status based on the IOTF criteria [16, 17] at age 3 years.

Visual description	Child’s actual weight status, n (%)			Kappa, κ
	Very underweight/ Underweight	Normal	Overweight/ Obesity	
	(n = 161)	(n = 591)	(n = 69)	
Underweight	18 (11.2)	34 (5.8)	0 (0.0)	
Normal	137 (85.1)	539 (91.2)	51 (73.9)	0.134
Overweight	6 (3.7)	18 (3.0)	18 (26.1)	

n = number; % = percentage

In our supplementary analyses using the WHO standards for actual weight classification, poor agreements between perceived and actual weight status were also found for both verbal description (κ = 0.100) (S2 Table) and visual description (κ = 0.147) (S3 Table).

### Differences in verbal and visual descriptions

The percentages of children whose weight were accurately described by their mothers were almost identical for verbal (n = 577, 70.3%) and visual descriptions (n = 575, 70.0%). In verbal description, the prevalence of underestimation (n = 147, 17.9%) was higher than that of overestimation (n = 97, 11.8%). In contrast, the prevalence of overestimation (n = 161, 19.6%) was higher than that of underestimation (n = 85, 10.4%) in visual description.

As shown in Table 3, in both verbal and visual description tasks, more than half (n = 468, 57.0%) of the mothers were able to accurately describe their child’s weight status, while 9.6% (n = 79) of the mothers consistently overestimated and 6.6% (n = 54) of the mothers consistently underestimated their child’s weight status. About a quarter (n = 220, 26.8%) of the mothers were not consistent in describing their child’s weight status both verbally and visually.

**Table 3:** Comparison of accuracy between the mothers’ verbal and visual descriptions.

Verbal description	Visual description		
	Overestimation	Accurate	Underestimation
Overestimation	79	18	0
Accurate	78	468	31
Underestimation	4	89	54

### Factors associated with accuracy and consistency of weight perceptions

Table 4 shows the factors associated with consistent and inconsistent perception of child’s weight status using verbal and visual descriptions. Higher maternal BMI at 18 months postpartum was observed in mothers who consistently underestimated their child’s weight status both verbally and visually. The weight status of first-born children was more likely to be consistently underestimated by their mothers compared to children who have elder sibling(s). Of the four groups of mothers categorized according to consistency of weight perceptions, children who were consistently overestimated had the lowest BMI while children who were consistently underestimated had the highest BMI. The weight status of IVF children were also more likely to be inconsistently perceived by their mothers.

**Table 4:** Factors associated with consistent and inconsistent misperception of child’s weight status using both verbal and visual descriptions.

Variables	Consistently accurate	Consistent overestimation	Consistent underestimation	Inconsistent perception	P value
	(n = 468)	(n = 79)	(n = 54)	(n = 220)	
Types of conception (n, %)					0.020
Natural	434 (92.7)	78 (98.7)	52 (96.3)	196 (89.1)	
IVF	34 (7.3)	1 (1.3)	2 (3.7)	24 (10.9)	
Ethnicity (n, %)					0.452
Chinese	272 (58.2)	44 (55.7)	27 (50.0)	124 (56.4)	
Malay	124 (26.6)	17 (21.5)	17 (31.5)	52 (23.6)	
Indian	71 (15.2)	18 (22.8)	10 (18.5)	44 (20.0)	
Marital status (n, %)					0.920
Single/divorced	14 (3.0)	1 (1.3)	1 (1.9)	7 (3.2)	
Married	446 (97.0)	77 (98.7)	53 (98.1)	210 (96.8)	
Mother’s education (n, %)					0.564
None/primary/secondary	121 (26.1)	24 (30.8)	18 (33.3)	70 (32.0)	
Post-secondary	161 (34.8)	30 (38.5)	18 (33.3)	74 (33.8)	
Tertiary	181 (39.1)	24 (30.8)	18 (33.3)	75 (34.2)	
Monthly household income (n, %)					0.047
≤1999	62 (14.1)	11 (14.7)	8 (16.3)	32 (15.7)	
2000–5999	218 (49.7)	41 (54.7)	33 (67.3)	118 (57.8)	
≥6000	159 (36.2)	23 (30.7)	8 (16.3)	54 (26.5)	
Child’s gender (n, %)					0.555
Male	242 (51.7)	47 (59.5)	26 (48.1)	114 (51.8)	
Female	226 (48.3)	32 (40.5)	28 (51.9)	106 (48.2)	
Child’s weight status at 3 years old (n, %)					<0.001
Underweight	10 (2.1)	77(97.5)	0 (0.0)	74 (33.6)	
Normal	455 (97.2)	2 (2.5)	12 (22.2)	122 (55.5)	
Overweight	3 (0.6)	0 (0.0)	42 (77.8)	24 (10.9)	
Birth order (n, %)					<0.001
1	216 (46.2)	19 (24.1)	30 (55.6)	107 (48.6)	
≥2	252 (53.8)	60 (75.9)	24 (44.4)	113 (51.4)	
Mother’s BMI at 18 months post-delivery (kg/m^2^)	23.84 (4.62)^‡^	23.27 (5.16)^‡^	26.72 (6.51)^#,†,§^	24.10 (4.70)^‡^	0.004
Mother’s age (years)	31.06 (5.19)	30.67 (4.34)	30.41 (5.08)	31.27 (5.04)	0.630
Child’s BMI at 3 years old (kg/m^2^)	15.85 (0.86)^†,‡^	14.13 (0.80)^#,‡,§^	18.22 (2.08)^#,†,§^	15.53 (2.13)^†,‡^	<0.001

BMI = body mass index, IVF = *in vitro* fertilization; kg = kilogram; m = metre

Post-hoc tests significantly different from consistent accurate^#^, consistent overestimation^†^, consistent underestimation^‡^ and inconsistent perception^§^ (*p*<0.05).

## Discussion

About 30% of Singaporean mothers in this study were unable to accurately describe their child’s weight status verbally or visually. The mothers were more likely to underestimate their child’s weight status using verbal description and to overestimate their child’s weight status using visual description. Importantly, many mothers of underweight children over-estimated, and many mothers of overweight and obese children under-estimated, their child’s weight status. In contrast, significantly fewer mothers of normal-weight children were inaccurate. These findings reinforced the significance of recognizing maternal misperception of their child’s weight as a public health issue that urgently needs to be tackled [19]. We also identified birth order, maternal BMI and child’s weight status to be significantly associated with consistently inaccurate verbal and visual descriptions.

In this study, overweight and obese children were more likely to be under-estimated by their mothers. This finding is consistent with previous studies conducted in western countries [12]. Our study added that very underweight and underweight children were more likely to be over-estimated by their mothers. There are several possible explanations for mothers of underweight or overweight children to describe their child’s weight status as normal. These mothers may be reluctant to stigmatize their child as underweight or overweight [20]. They may believe that they were able to control their child’s diet and maintain their child at normal weight. They may be unwilling to change their current feeding practices. They may lack knowledge and awareness about the definition of healthy weight in young children. They may have labeled their child as normal weight because their child was happy, not sick and could perform daily activities [21]. The current obesogenic environment in Singapore may have created a scenario in which the overweight status is common and thus, perceived as normal [22].

The high prevalence of misperceptions in Singapore is alarming, especially among the mothers of children with unhealthy weights, because mothers’ perceptions can affect their child feeding practices. It has been reported that parents who perceived their child as underweight or normal tended to increase feeding their child under emotional distress or use food as a reward, compared to those who perceived their child as overweight [11]. Such feeding practices could lead to excessive weight gain [11]. On the contrary, parents who misperceived their underweight child as heavier may restrict their child’s food intake, resulting in nutrient deficiency, impaired growth and delayed development. Parental misperceptions of both overweight and underweight children are therefore detrimental to their child’s health status.

Our study showed that Singaporean mothers were more likely to underestimate their child’s weight status using verbal description rather than visual description. Similar finding was also reported in the USA by Eckstein et al. [23] but not in Mexico by Souto-Gallardo et al. [24]. Eckstein et al.[23] concluded that visual description were superior to verbal description, while Souto-Gallardo et al.[24] found no difference between visual and verbal descriptions by parents. In the present study, the difference in maternal accuracy between verbal and visual descriptions that we have found most likely exists in the cohort and is not an artifact arising from our methodology. Quite in the contrary, this observation was made possible by the way we administered the visual description task. We presented the TSS sketches to the mothers one-by-one in a random fashion so that the mothers could not make direct visual comparison of the sketches lined up in an increasing weight scale from lightest to heaviest. In this way, how the mothers *visually* perceived their child's weight status is accurately depicted by a particular sketch shown that they decided to select out of all the other sketches that were shown one-by-one and separately from the selected sketch. If all the sketches were presented in ascending weights, there may be a tendency for the mothers to choose a particular sketch based on how they *verbally* perceived their child’s weight status.

The TSS was previously used to assess maternal perception of toddler’s weights in Baltimore, Maryland, USA, where inaccuracy was found to be as high as 70% [25]. While the overall inaccuracy of 30% in the present study was much lower, the difference is most likely due to the lower rate of overweight and obese children (8.4%) in Singapore compared to Baltimore (29.2%) [25]. Had the obesity rates in this study been higher, we would have found more inaccuracy because 73.9% of the mothers of overweight and obese children did under-estimate their child’s weight status by visual description. Other explanations for the discrepancy in findings between study in Baltimore and ours may be the differences in socioeconomic status including marital status, household income and education level, as well as mother’s weight status. Since Hager et al. [25] had used ethnicity-neutral sketches in the TSS, we are confident that the TSS is a valid visual description tool for our study even though it was not originally developed for use to assess mothers of Asian children.

The weights of first-born children tended to be consistently under-estimated by Singaporean mothers. This may be due to the lack of parenting experience, which has become more common as family size decreases in developed countries. Maternal postpartum weight status also influenced mother’s perception of their child’s weight. Thinner mothers tended to over-estimate their child’s weight status, an observation that was also made by Warschburger and Kröller [26]. In contrast, heavier mothers tended to under-estimate the weight of their heavier child. Health promotion efforts should therefore focus on these groups of mothers who are particularly at risk of misperceptions.

The strengths of this study include measurements of the children by a team of trained and regularly audited research staff using international standards, hence increasing the reliability of the children’s actual weight status. The international standards are linked to the widely accepted adult cutoff points [16, 17], allowing us to estimate more precisely the prevalence of maternal misperception of child’s weight status. We used a validated tool, the TSS [18], to assess the mothers’ visual perception. However, this study presents a few limitations. The mothers may not be the primary caregivers of their child because many Singaporean women hold full-time jobs. We did not evaluate how the mothers perceived their own weight status. The sample size for underweight and overweight or obese children was small, suggesting that further studies in Singapore should include larger sample size. Also, we did not measure children’s body composition to substantiate their anthropometric measurements. Finally, several differences in characteristics between the excluded and included study participants were noted and thus our findings cannot be generalized to Singaporean mothers who were single, younger and have lower educational level and household income.

In conclusion, misperception of child’s weight status is prevalent among Singaporean mothers, especially among mothers of overweight and underweight young children. As such, paediatricians, general physicians and healthcare professionals must not rely on self-reported information from mothers about their child’s weight status. Instead, accurate anthropometric measurements of all children attending child health clinics should always be taken and used to educate their parents and caregivers. In this way, any misperceptions by mothers in the weights of their young child can be rectified in a timely manner. If the observations in this cohort of Singaporean mothers are replicated in future studies involving mothers of Chinese, Malay or Indian ethnicities, they offer enormous implications for the global prevention of childhood obesity because these three ethnicities constitute a considerably large proportion of the total population worldwide.

## Supporting Information

S1 Table Comparison of maternal and child characteristics between the GUSTO participants who were included and excluded from this study. (DOCX)

S2 Table The agreement between mother’s verbal description of the child’s perceived weight status and the child’s actual weight status based on the WHO standards at age 3 years. (DOCX)

S3 Table The agreement between mother’s visual description of the child’s perceived weight status and the child’s actual weight status based on the WHO standards at age 3 years. (DOCX)
