# Supplementary material for: Singaporean Mothers’ Perception of Their Three-year-old Child’s Weight Status: A Cross-Sectional Study
Source: PLoS One. 2016 Jan 28;11(1):e0147563. doi: 10.1371/journal.pone.0147563 (PMC4731472; doi:10.1371/journal.pone.0147563)
Supplement: S1 Table — (DOCX) [file pone.0147563.s001.docx]

| **S1 Table. Comparison of maternal and child characteristics between the GUSTO participants who were included and excluded from this study.** | | | |
| --- | --- | --- | --- |
|  | Excluded  (n=416) | Included  (n=821) | P value |
| Types of conception (n, %) |  |  | 0.341 |
| Natural | 392 (94.2) | 760 (92.6) |  |
| IVF | 24 (5.8) | 61 (7.4) |  |
| Child’s weight status (n, %) |  |  | 0.845 |
| Very underweight | 1 (0.9) | 11 (1.3) |  |
| Underweight | 20 (18.7) | 150 (18.3) |  |
| Normal | 78 (72.9) | 591 (72.0) |  |
| Overweight | 7 (6.5) | 45 (5.5) |  |
| Obesity | 1 (0.9) | 24 (2.9) |  |
| Child’s gender (n, %) |  |  | 0.522 |
| Male | 190 (54.4) | 429 (52.3) |  |
| Female | 159 (45.6) | 392 (47.7) |  |
| Ethnicity (n, %) |  |  | 0.575 |
| Chinese | 224 (53.8) | 467 (56.9) |  |
| Malay | 112 (26.9) | 210 (25.6) |  |
| Indian | 80 (19.2) | 144 (17.5) |  |
| Mother’s education (n, %) |  |  | 0.002 |
| None/primary/secondary | 148 (36.5) | 233 (28.6) |  |
| Post-secondary | 148 (36.5) | 283 (34.8) |  |
| Tertiary | 110 (27.1) | 298 (36.6) |  |
| Marital status (n, %) |  |  | 0.049 |
| Single/divorced | 21 (5.2) | 23 (2.8) |  |
| Married | 381 (94.8) | 786 (97.2) |  |
| Household monthly income (n, %) |  |  | 0.006 |
| <2000 | 68 (17.7) | 113 (14.7) |  |
| 2000-5999 | 230 (59.7) | 410 (53.5) |  |
| ≥6000 | 87 (22.6) | 244 (31.8) |  |
| Birth order 9n, %) |  |  | 0.749 |
| 1 | 162 (46.4) | 372 (45.3) |  |
| ≥2 | 187 (53.6) | 449 (54.7) |  |
| Mother’s BMI at 18 months post-delivery (kg/m^2^) | 24.44 (4.71) | 24.02 (4.86) | 0.356 |
| Mother’s age (years) | 29.67 (5.25) | 31.04 (5.07) | <0.001 |
| Child’s BMI at 3 years old (kg/m^2^) | 15.81 (1.36) | 15.75 (1.63) | 0.733 |
| BMI, body mass index, IVF, *in vitro* fertilization; kg, kilogram; m, metre | | | |
