# Supplementary material for: Singaporean Mothers’ Perception of Their Three-year-old Child’s Weight Status: A Cross-Sectional Study
Source: PLoS One. 2016 Jan 28;11(1):e0147563. doi: 10.1371/journal.pone.0147563 (PMC4731472; doi:10.1371/journal.pone.0147563)
Supplement: S2 Table — (DOCX) [file pone.0147563.s002.docx]

| **S2 Table. The agreement between mother’s verbal description of the child’s perceived weight status and the child’s actual weight status based on the WHO standards [18] at age 3 years.** | | | | | | |
| --- | --- | --- | --- | --- | --- | --- |
| Verbal description | Child’s actual weight status based on the WHO, n (%) | | | | | Kappa, κ |
|  | Very underweight  (n=2) | Underweight  (n=15) | Normal  (n=672) | Overweight  (n=112) | Obesity  (n=20) |  |
| Very underweight | 0 (0.0) | 0 (0.0) | 6 (0.9) | 0 (0.0) | 0 (0.0) | 0.100 |
| Underweight | 1 (50.0) | 13 (86.7) | 151 (22.5) | 0 (0.0) | 0 (0.0) |  |
| Normal | 1 (50.0) | 2 (13.3) | 505 (75.1) | 95 (84.8) | 2 (10.0) |  |
| Overweight | 0 (0.0) | 0 (0.0) | 10 (1.5) | 17 (15.2) | 17 (85.0) |  |
| Very overweight | 0 (0.0) | 0 (0.0) | 0 (0.0) | 0 (0.0) | 1 (5.0) |  |
