# Supplementary material for: Singaporean Mothers’ Perception of Their Three-year-old Child’s Weight Status: A Cross-Sectional Study
Source: PLoS One. 2016 Jan 28;11(1):e0147563. doi: 10.1371/journal.pone.0147563 (PMC4731472; doi:10.1371/journal.pone.0147563)
Supplement: S3 Table — (DOCX) [file pone.0147563.s003.docx]

| **S3 Table. The agreement between mother’s visual description of the child’s perceived weight status and the child’s actual weight status based on the WHO standards [18] at age 3 years.** | | | | |
| --- | --- | --- | --- | --- |
| Visual description | Child’s actual weight status based on the WHO, n (%) | | | |
|  | Very underweight / Underweight  (n=17) | Normal  (n=672) | Overweight/ Obesity  (n=132) | Kappa, κ |
| Underweight | 5 (29.4) | 45 (6.7) | 2 (1.5) | 0.147 |
| Normal | 11 (64.7) | 608 (90.5) | 108 (81.8) |  |
| Very overweight | 1 (5.9) | 19 (2.8) | 22 (16.7) |  |
